# Modulating spectral response of raw photosynthetic pigments via ternary cadmium chalcogenide quantum dots: simultaneous enhancement at green spectrum and inhibition at UV region

**DOI:** 10.1007/s11120-024-01085-7

**Published:** 2024-02-26

**Authors:** Sümeyye Aykut, Nida Ük, İbrahim Yağız Coşkun, Sultan Şahin Keskin, Ilgın Nar, Levent Trabzon, Caner Ünlü

**Affiliations:** 1 Department of Chemistry, Faculty of Science and Letters, Istanbul Technical University, 34469 Maslak, Istanbul Turkey; 2 Polymer Science and Technology, Istanbul Technical University, 34469 Maslak, Istanbul Turkey; 3 Department of Nanoscience and Nanoengineering, Istanbul Technical University, 34469 Maslak, Istanbul Turkey; 4 MEMS Research Center, Istanbul Technical University, Istanbul, Turkey; 5 Istanbul Technical University Nanotechnology Research and Application Center (ITUNano), Istanbul, Turkey; 6 Faculty of Mechanical Engineering, Istanbul Technical University, Istanbul, Turkey

**Keywords:** Photosynthesis, Chlorophyll fluorescence, Quantum dots

## Abstract

Photosynthesis relies on the absorption of sunlight by photosynthetic pigments (PPs) such as chlorophylls and carotenoids. While these pigments are outstanding at harvesting light, their natural structure restricts their ability to harvest light at specific wavelengths. In this study, Oleic acid-capped CdSeS and CdTeS ternary quantum dots (QDs) were synthesized using a novel two-phase synthesis method. Then, these QDs were used to interact with raw PPs, a mixture of chlorophylls and carotenoids isolated from spinach. Our findings revealed the following: (1) Interacting QDs with raw PPs effectively inhibited the chlorophyll fluorescence of the pigments upon excitation in UV light region (250–400 nm) without causing any damage to their structure. (2) By forming an interaction with QDs, the chlorophyll fluorescence of raw PPs could be induced through excitation with green-light spectrum. (3) The composition of the QDs played a fundamental role in their interaction with PPs. Our study demonstrated that the photophysical properties of isolated PPs could be modified by using cadmium-based QDs by preserving the structure of the pigments themselves.

## Introduction

Photosynthesis, which is responsible for sustaining aerobic life on Earth, relies on the absorption of sunlight by photosynthetic pigments (PPs) like chlorophylls and carotenoids (Croce and Van Amerongen 2014, 2020). While these pigments excel at capturing light, their natural structure limits their absorption capacity to specific wavelengths (Croce and Van Amerongen 2014, 2020). The primary PPs in higher plants, chlorophylls and carotenoids, absorb light predominantly in the blue region (400–500 nm) and near-infrared region (620–720 nm), allowing for efficient photosynthesis within these ranges (Croce and Van Amerongen 2014, 2020). However, due to the presence of carbon–carbon and carbon–oxygen double bonds, chlorophylls and carotenoids also absorb light in the harmful UV spectrum (200–350 nm), posing risks to biomolecules (Croce and Van Amerongen 2014, 2020). The detrimental effects of UV light on biomolecules intensify with prolonged exposure, making sunlight or white light exposure a concern (Croce and Van Amerongen 2014, 2020).

Plants and algae possess protective mechanisms, such as non-photochemical quenching, to mitigate the effects of high-intensity light (Dall’Osto et al. 2014; Ünlü et al. 2014; Fox et al. 2018). This mechanism converts excess absorbed light into heat and dissipates it, safeguarding organisms against the harmful effects of reactive oxygen species (Dall’Osto et al. 2014; Ünlü et al. 2014; Fox et al. 2018). However, when chlorophylls and carotenoids are isolated from living organisms, these protective mechanisms no longer function, making the biomolecules prone to degradation under harsh environmental conditions, including exposure to UV radiation and high temperatures (Salama et al. 2011; Piccini et al. 2020; Jovanić et al. 2022). Despite their natural light-harvesting capabilities, the delicate structure of chlorophylls and other PPs limits their practical use in ex vivo applications, such as solar cells, LEDs, and biosensors.

The long-term stability of biomolecules outside their natural environment presents a significant challenge that hinders their utilization in ex vivo applications. Exposure to UV light is a key factor contributing to biomolecule degradation (Salama et al. 2011; Piccini et al. 2020; Jovanić et al. 2022). Many biomolecules, including chlorophylls and carotenoids, possess carbon–carbon or carbon–oxygen double bonds, which absorb UV light in the range of 200–350 nm. Ultraviolet B (UV-B, 280–315 nm) and Ultraviolet C (UV-C, 100–280 nm) radiation specifically causes degradation by breaking these double bonds (Salama et al. 2011; Piccini et al. 2020; Jovanić et al. 2022). Consequently, employing biomolecules under natural sunlight, which encompasses the UV light spectrum, becomes problematic (Salama et al. 2011; Piccini et al. 2020; Jovanić et al. 2022).

Photosynthetic pigments have been widely studied in a crucial ex vivo application in solar cell development (Syafinar et al. 2015; Bhogaita et al. 2016; Ramanarayanan et al. 2017; Chandra Maurya et al. 2019; Kabir et al. 2019). The photosynthetic pigments generally extracted through ethanol extraction, which is easy, inexpensive and relatively non-toxic (Syafinar et al. 2015; Bhogaita et al. 2016; Ramanarayanan et al. 2017; Chandra Maurya et al. 2019; Kabir et al. 2019; Ferreira et al. 2021). Mainly, chlorophylls extracted from various plant sources, notably spinach and red amaranth, emerge as key sensitizers. Syafinar et al. delved into the absorption characteristics of chlorophyll extracted from spinach using different solvents, including ethanol and water, and discussed possibility of using photosynthetic pigments in solar cell development (Syafinar et al. 2015). Then, Kabir et al. emphasized the potential of natural green dye extracted from spinach for solar cells, although its measured cell efficiency remains modest at 0.398%, raising concerns about stability (Kabir et al. 2019). Meanwhile, Maurya et al. focused on a natural dye from Cassia fistula flowers, utilizing ethanol as a solvent, provided insight into the molecular structure and electron transfer characteristics and discussed possibility of using raw photosynthetic pigments in the development of solar cells (Chandra Maurya et al. 2019). Furthermore, red amaranth took center stage, with chlorophyll as a dominant sensitizer showing superior performance in terms of photocurrent density (1.3 mA/cm^2^) and energy conversion efficiency (0.53%) (Ramanarayanan et al. 2017). Also, a broader review underscored the significance of chlorophyll, along with other plant pigments like anthocyanin, betalain, and carotenoids, in hybrid solar cells (Bhogaita et al. 2016). Despite challenges related to pH sensitivity and stability, the eco-friendly nature of natural dyes, especially raw photosynthetic pigments, positioned them as promising candidates for sustainable and efficient solar energy conversion (Syafinar et al. 2015; Bhogaita et al. 2016; Ramanarayanan et al. 2017; Chandra Maurya et al. 2019; Kabir et al. 2019).

Quantum dots (QDs), which are fluorescent semiconductor nanocrystals with a diameter smaller than the Bohr-exciton diameter, have found applications in various studies related to natural photosynthesis (Schmitt et al. 2012; Lukashev et al. 2016; Budak et al. 2020; Liu et al. 2021; Parrish et al. 2021; Ünlü et al. 2022). The unique photophysical properties of QDs have made them a subject of interest for investigating their interaction with isolated photosystems or components of photosystems, such as reaction centers and light-harvesting complexes. Since the early 2000s, researchers have been exploring the potential of QDs as energy donors to enhance light-harvesting (Schmitt et al. 2012; Lukashev et al. 2016; Budak et al. 2020; Liu et al. 2021; Parrish et al. 2021; Ünlü et al. 2022). Particularly, cadmium-based QDs (such as CdTe, CdSe/ZnS, CdSe) capped with polymeric ligands or thiol-carboxylate-based ligands have been considered promising energy donors for light-harvesting complexes isolated from plants, purple bacteria, microalgae, and other organisms (Schmitt et al. 2012; Lukashev et al. 2016; Budak et al. 2020; Liu et al. 2021; Parrish et al. 2021; Ünlü et al. 2022). However, despite many studies having been done to understand the effect of the presence of quantum dots on isolated systems like photosystems or light-harvesting complexes, there hasn't been any study conducted on the interaction between raw PPs (isolated crude PPs without any further purification/isolation) and cadmium-based QDs, which could provide valuable insights into the fundamental interaction between intact chlorophyll-carotenoid complexes and QDs.

In our study, we synthesized oleic acid-capped CdSSe and CdSTe ternary QDs using a novel two-phase synthesis method. These QDs were then utilized to interact with raw PPs, which consisted of a mixture of chlorophylls and carotenoids isolated from spinach. Our findings revealed the following: (1) Conjugation of QDs with raw PPs effectively decreased the spectral response of PPs upon excitation with UV light, without causing any damage to their structural integrity. (2) By forming a conjugate with QDs, the spectral response of raw PPs could be extended to include the green-light spectrum. (3) The composition of the QDs played a fundamental role in the interaction with PPs. Our study demonstrated that the photophysical properties of isolated PPs could be modulated by employing cadmium-based QDs, all while preserving the intact structure of the pigments themselves.

## Materials and methods

### Chemicals

Cadmium acetate, myristic acid, oleic acid, selenium powder and tellurium powder were purchased from Sigma-Aldrich Co. Sodium borohydride, thiourea, 3-mercaptopropionic acid and sodium hydroxide were purchased from Merck.

### Synthesis of cadmium myristate

Cadmium myristate (Cd-Mry) was synthesized by dissolving cadmium acetate and myristic acid in methanol at room temperature in a 1:3 mol ratio, respectively. Myristic acid (0.003 mmol) was dissolved in 100 mL of methanol with 2.500 mmol of NaOH, and 0.001 mmol of cadmium acetate was dissolved in 5 mL of methanol. Once the two solutions became homogeneous, the cadmium acetate solution was added drop by drop (approximately 1 drop/s) to the myristic acid solution in a large Erlenmeyer flask. Finally, the resulting precipitate was filtered using filter paper and dried at room temperature.

### Synthesis of NaHTe

Following a method outlined in the literature (Ünlü 2019; Kestir et al. 2023), 0.4 mol of NaBH_4_ was dissolved in 10 mL of distilled water under N_2_ gas. A glass syringe was used to add saturated distilled water to 0.08 mmol of Te, which had been degassed with N_2_ atmosphere in a beaker for 15 min. The reaction was completed in 15 min at 60 °C, indicated by a color change in the solution from dark brown to lilac.

### Synthesis of NaHSe

A method described in the literature (Ünlü 2019; Erkan et al. 2023) was followed. A NaHSe solution was prepared by following the same instructions as the NaHTe synthesis. 0.134 mmol of Se was kept under N_2_ gas in a flask for 15 min. Simultaneously, 0.4 mol of NaBH_4_ was dissolved in 10 mL of distilled water and cautiously transferred to the Se flask via a glass syringe after waiting in an N_2_ atmosphere. The reaction occurred in 15 min at 60 °C.

### Synthesis of oleic acid capped CdTeS QDs

Te-rich CdTeS QDs were synthesized using the liquid–liquid interface method (Kestir et al. 2023). The organic liquid phase was prepared by slowly adding 5 mmol of oleic acid drop by drop to 0.111 g of cadmium myristate (Cd-Mry) in 50 mL of toluene. The mixture was stirred under N_2_ gas for 15 min at 90 °C. For the water liquid phase, 0.8 mmol thiourea was dissolved in 45 mL distilled water in a three-necked flask under N_2_. Then, 0.08 mmol NaHTe solution was added with a syringe to prevent oxidation after the thiourea solution was saturated with N2 gas. QD formation was initiated as the two liquid phases were combined by adding Cd-Mry and oleic acid in toluene to the three-necked flask under a N_2_ atmosphere at 100 °C. The organic phase (1–2 mL) was periodically collected after starting the formation to examine QDs under UV light. The synthesis was terminated at various times, and QDs were kept at 0–2 °C after precipitation in methanol (1:1, volume) for characterization. S-rich CdTeS QDs were synthesized by increasing thiourea to 2 mmol, while CdSeS QDs were synthesized by replacing NaHTe with NaHSe, using the same amounts (Erkan et al. 2023).

### Interaction of PPs with QDs

Pigments (PPs) were extracted by submerging approximately 2.0 g of spinach leaves in 40 mL of ethanol at a temperature of 0–2 °C for 48 h. Afterward, the pigments were filtered using filter paper and dried in a fume hood. Once dried, the pigments were dissolved in 10 mL of chloroform to prepare them for interactions. For QD solutions, QDs (approximately 5.0 mg each) were dissolved in 10 mL of chloroform. These solutions were then centrifuged at 14,000 rpm for 2 min to remove any unwanted large clusters. To facilitate the interaction between the PPs and QDs, the solutions were mixed in a 50 ml Erlenmeyer flask and shaken in the dark for 1 h at 400 rpm. To make a comparison, separate solutions of pure QDs and PPs with the exact same concentration as the PP-QD mixtures were prepared and treated in the same manner. Finally, 3 ml aliquots were collected from each mixture, and further spectroscopic measurements were conducted.

### Structural and optical characterization

The crystal structure of Cd-based quantum dots (QDs) was analyzed using an RIGAKU SmartLab X-ray diffraction spectrometer with Cu Kα radiation at a wavelength of 1.5406 Å and a voltage of 30 kV. The QDs were measured in the 2-theta range between 15° and 70° with a step size of 0.01° and a duration of 4 s for each measurement.

The surface properties of the quantum dots were characterized using Attenuated Total Reflectance FTIR (ATR-FTIR) spectroscopy. The FTIR spectra of the quantum dots were recorded in the range of 700–4000 cm^−1^ using a Perkin Elmer ATR-FTIR Spectrophotometer.

For characterizing optical properties, the absorption spectrum of the QDs was measured using a Varian Cary double-beam ultraviolet–visible (UV–vis) spectrophotometer. Additionally, a Varian Cary Eclipse Fluorescence Spectrofluorometer was used to record the excitation and emission spectra of each sample to understand their emission properties. To avoid self-absorption that may arise due to the presence of QDs, the optical density of each sample type was adjusted to 0.1 at 350 nm before collecting the emission and excitation spectra. The emission spectrum was collected at several different excitation wavelengths, while the excitation spectrum was collected at two different emission wavelengths: 685 nm and 720 nm.

The quantum yield of each quantum was calculated using Coumarin 102 as a reference, following the methodology described in the literature (Erkan et al. 2023). All measurements were conducted at room temperature.

## Results

Oleic acid-capped cadmium chalcogenide QDs were synthesized using the two-phase synthesis method, a traditional bottom-up approach. Three different compositions were targeted: CdSeS, Te-rich CdTeS, and S-rich CdTeS. Structural characterization of the Cd chalcogenide QDs involved X-ray diffraction (XRD) and Fourier-transform infrared (FTIR) measurements.

**Fig. 1:**
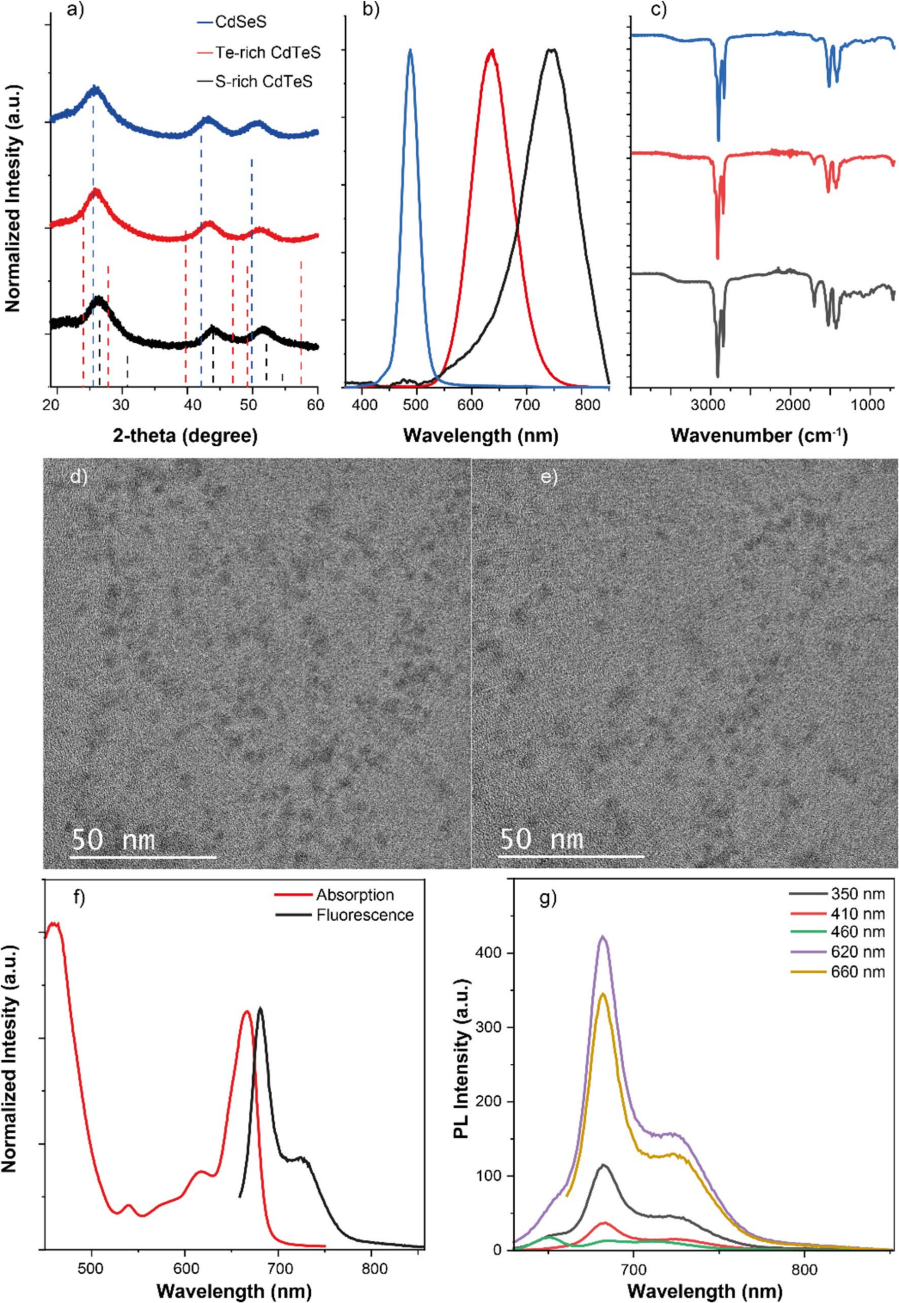
**a** XRD spectrum of CdSeS, Te-rich CdTeS and S-rich CdTeS QDs. The dotted lines represent XRD spectrum of cubic bulk CdS (black), bulk CdSe (blue) and bulk CdTe (red). **b** Fluorescence spectrum of CdSeS, Te-rich CdTeS and S-rich CdTeS QDs (excitation wavelength: 350 nm) **c** FTIR spectrum of CdSeS, Te-rich CdTeS and S-rich CdTeS QDs. TEM images of **d** CdTeS and **e** CdSeS QDs **f** Absorption and fluorescence spectrum (excitation wavelength: 660 nm) of raw photosynthetic pigments **g** Fluorescence spectrum of raw photosynthetic pigments at various excitation wavelengths

The XRD analysis was conducted to determine the composition and crystal structures of the QDs (Fig. 1). It revealed that all three types of QDs exhibited a cubic crystal structure, as evidenced by three prominent broad peaks corresponding to the Miller indices [111], [220], and [311] (Fig. 1a). The specific XRD peak positions for each QD composition were as follows (Fig. 1); CdSeS: [111]: 25.63, [220]: 43.14, [311]: 50.80, Te-rich CdTeS: [111]: 25.86, [220]: 43.30, [311]: 51.45, S-rich CdTeS: [111]: 26.23, [220]: 43.90, [311]: 51.69. Additionally, the full-width at half maximum (FWHM) of the XRD peak corresponding to the [111] plane was determined. For CdSeS, the FWHM was 3.50 degrees, for Te-rich CdTeS, the FWHM was 3.20 degrees, and for S-rich CdTeS, the FWHM was 2.65 degrees (Fig. 1a).

The QDs exhibited distinct photophysical properties, each displaying a unique emission color (Fig. 1b). The CdSeS QDs emitted light with a single peak centered around 505 nm, accompanied by a FWHM of 40 nm. Meanwhile, the Te-rich CdTeS QDs displayed a single emission peak around 635 nm with an FWHM of 80 nm. Lastly, the S-rich CdTeS QDs emitted light with a single peak around 750 nm and had an FWHM of 90 nm (Fig. 1b).

Notably, all of the QDs demonstrated high photoluminescence quantum yields (QY). The QY of CdSeS QDs was calculated to be 85%, indicating efficient fluorescence emission. The Te-rich CdTeS QDs exhibited a QY of 54%, while the S-rich CdTeS QDs displayed a QY of 29%.

FTIR results indicated that the QDs shared similar organic groups on their surfaces, with carbon-hydrogen chains covering the QD surfaces (Fig. 1c). Notably, the FTIR peak corresponding to the -OH groups of oleic acid, typically observed around 3400–3600 cm^−1^, nearly disappeared in the FTIR spectra of oleic acid-capped QDs (Fig. 1c) (Caruntu et al. 2015; Masuku et al. 2021). In addition, peaks around 2800 cm^−1^ and 2900 cm^−1^ corresponding to CH_3_ bonds, a peak around 1700 cm^−1^ corresponding to C=O bonds and several peaks between 1500 and 1100 cm^−1^ corresponding to C=C and C–H bonds were observed clearly (Fig. 1c) (Caruntu et al. 2015; Masuku et al. 2021). Also, TEM images of QDs showed that size of CdSeS QDs was around 3 nm and size of CdTeS was around 4 nm (Fig. 1d and e).

PPs isolated from spinach exhibited the typical absorption and emission bands of chlorophyll. The absorption spectrum of these pigments displayed three peaks: a broad peak at 435 nm with a FWHM of 140 nm, a minor peak at 620 nm, and another major peak at 660 nm (Fig. 1f). The ratio of chlorophyll a: chlorophyll b: carotenoid was calculated as 8.6: 4: 1 using absorption spectrum of photosynthetic pigments in ethanol, as described in literature before (Lichtenthaler and Wellburn 1983). The emission behavior of the pigments varied depending on the excitation wavelength used. The emission spectrum of the pigments showed typical chlorophyll a + chlorophyll b emission characteristics; two overlapping peaks at 685 nm and 720 nm for excitation wavelengths of 350 nm, 410 nm and 660 nm. However, an extra peak emerged at 650 nm upon excitation with 460 nm and 620 nm. When collecting the photoluminescence excitation (PLE) spectrum of the pigments at an emission wavelength of 685 nm, two major bands were observed. One band covered a wide spectrum ranging from 225 to 470 nm, while the other band covered the spectrum between 500 and 700 nm, which consisted of a combination of several peaks (Fig. 1g).

The PPs isolated from spinach were combined with QDs in chloroform, and the interaction between the QDs and PPs was examined by collecting emission and excitation spectra for each sample. Initially, the emission spectrum of the QD-PP mixture was recorded and compared to the emission spectrum of PPs alone. For each sample, the excitation was performed using five different wavelengths: 350 nm, 410 nm, 460 nm, 620 nm, and 660 nm.

**Fig. 2:**
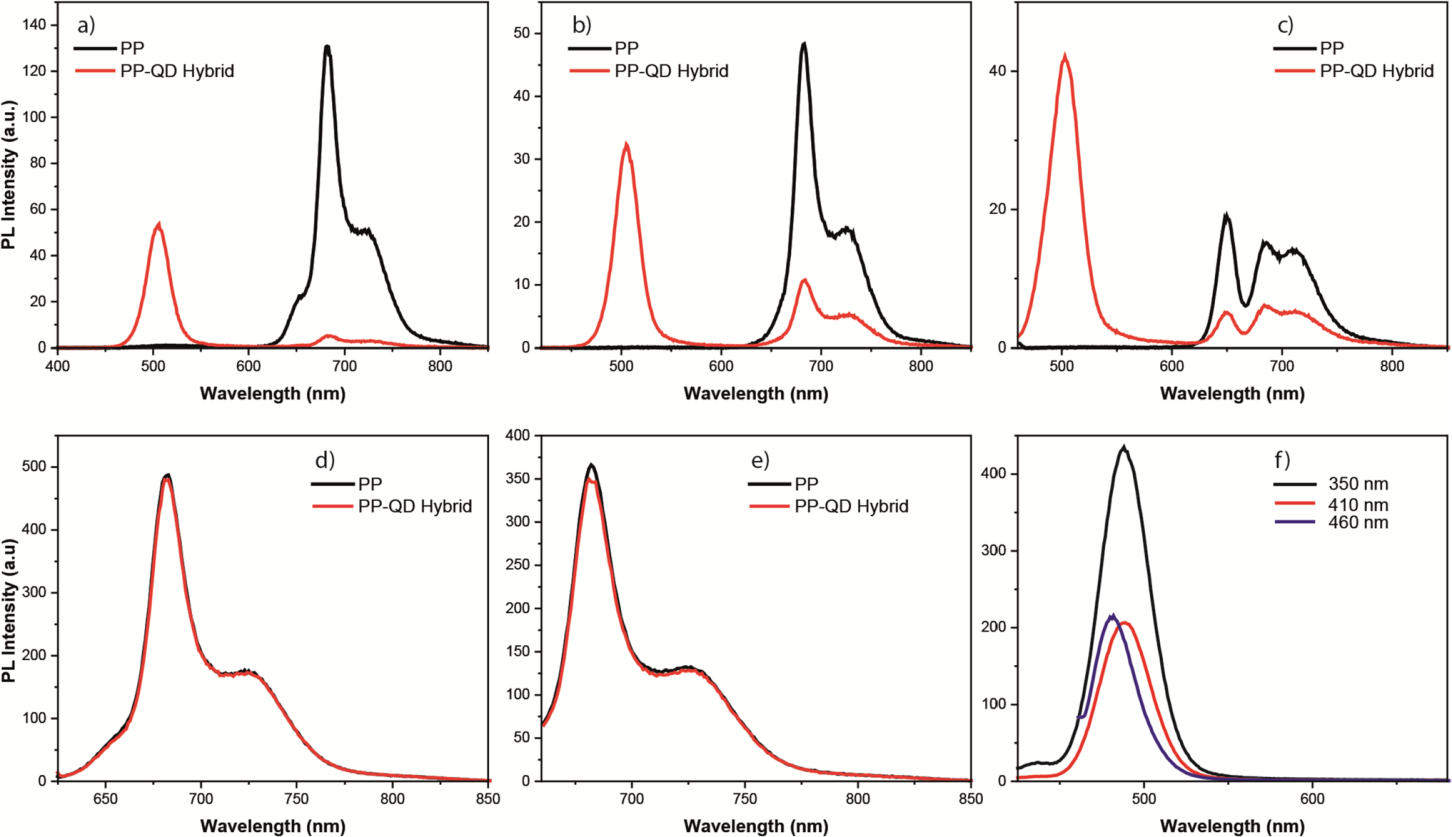
Fluorescence spectrum of photosynthetic pigments (PP) and CdSeS QD-PP hybrids at excitation wavelengths of **a** 350 nm, **b** 410 nm, **c** 460 nm, **d** 620 nm, and **e** 660 nm. **f** Fluorescence spectrum of CdSeS QD at various excitation wavelengths

Upon interaction with CdSeS QDs, it was observed that the emission of the PP was completely quenched when the QD-PP mixture was excited with 350 nm light (Fig. 2a). Conversely, the emission of CdSeS QDs was still observable, albeit with a 90% decrease in intensity (Fig. 2a). When excited with 410 nm light, the emission intensity of the PP decreased by 80%, while the emission intensity of the QD decreased by 95% in the QD-PP mixture (Fig. 2b). Similarly, upon 460 nm excitation, the emission intensity of the PP decreased by 75%, and the emission intensity of the QD decreased by 80% in the QD-PP mixture (Fig. 2c). However, upon 620 nm and 660 nm excitation, the emission of PP did not exhibit any significant difference in the QD-PP mixture (Fig. 2d and e). It should be noted that CdSeS QDs did not show any emission upon excitation at 620 nm and 660 nm (Fig. 2f).

**Fig. 3:**
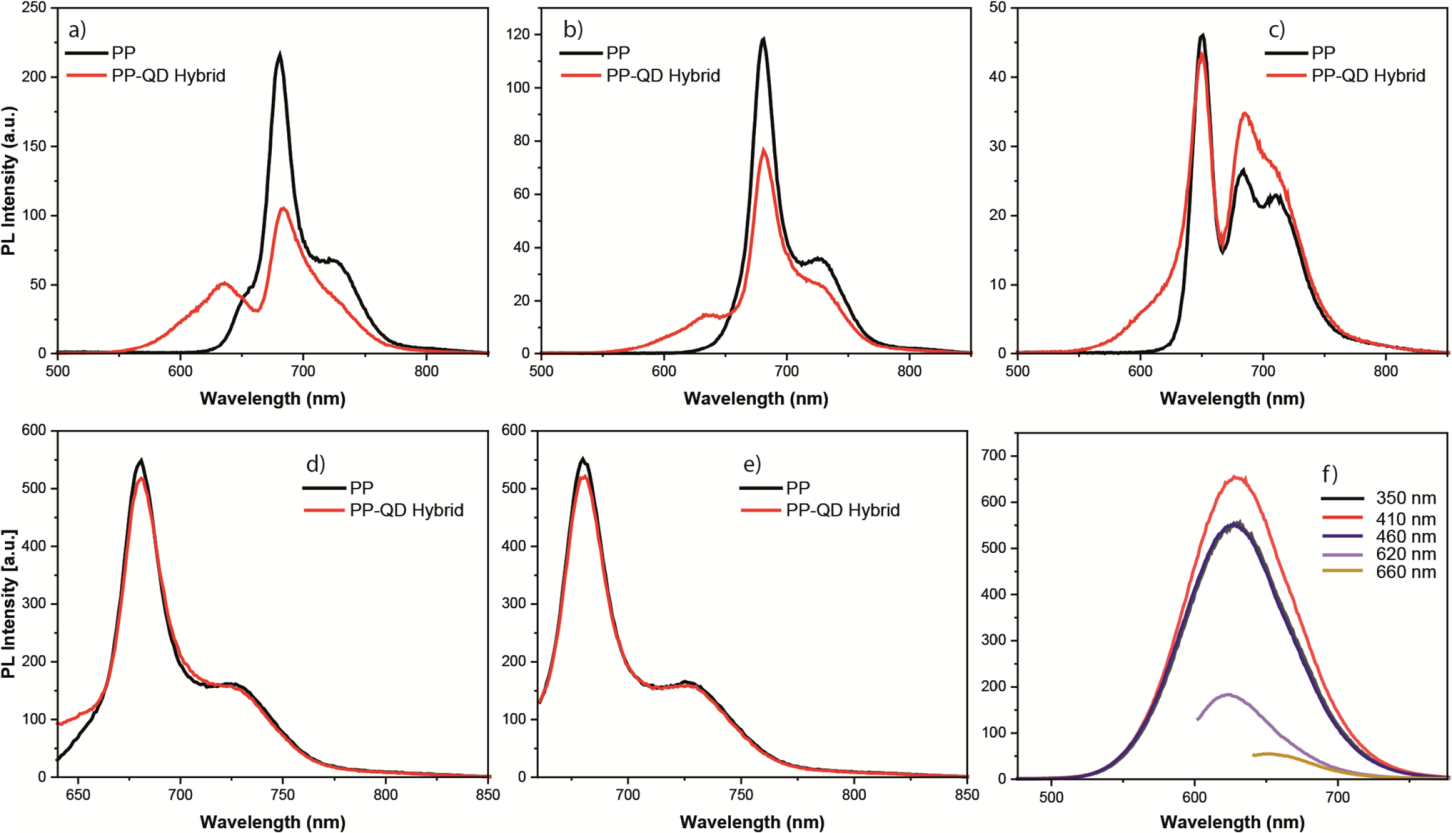
Fluorescence spectrum of photosynthetic pigments (PP) and Te-rich CdTeS QD-PP hybrids at excitation wavelengths of **a** 350 nm, **b** 410 nm, **c** 460 nm, **d** 620 nm, and **e** 660 nm. **f** Fluorescence spectrum of Te-rich CdTeS QD at various excitation wavelengths

As the PP interacted with Te-Rich CdTeS QDs, it was observed that the emission intensity of PP decreased by 65% upon excitation of the QD-PP mixture with 350 nm light (Fig. 3a). It should be noted that the emission spectra of the QDs and the PP overlapped in the range of 625 to 700 nm. The emission from Te-rich CdTeS QDs was still detectable at the peak wavelength of the QD emission spectrum (at 635 nm), albeit with a reduced intensity (decreased by 90%) (Fig. 3a). When excited with 410 nm light, the emission intensity of PP decreased by 50%. Similarly, the intensity of QD emission in the QD-PP mixture also decreased by 50% upon 410 nm excitation (Fig. 3b). Also, upon excitation at 620 nm and 660 nm, the emission of PP did not exhibit any significant differences in the QD-PP mixture (Fig. 3d and e).

**Fig. 4:**
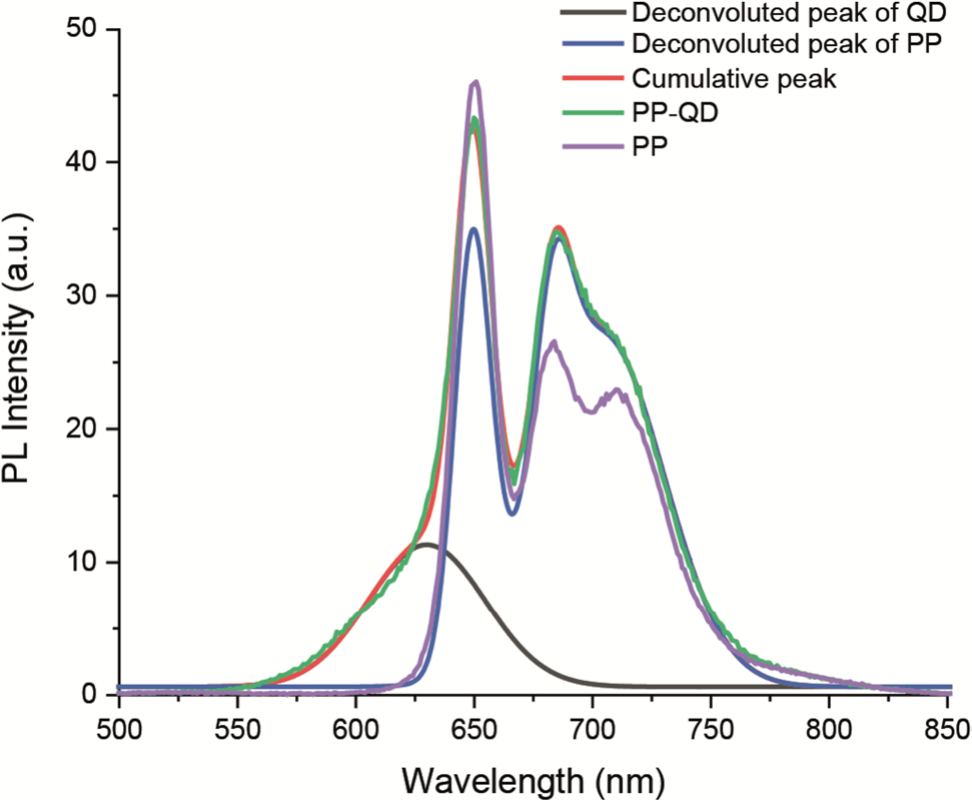
Fluorescence spectrum of photosynthetic pigments (PP) and Te-rich CdTeS QD-PP hybrids at an excitation wavelength of 460 nm. The fluorescence spectrum of Te-rich CdTeS QD-PP hybrids was deconvoluted to get rid of emission which arose from QDs

However, upon excitation at 460 nm, the intensity of emission of PP remained relatively unchanged at the first peak of the PP spectrum, around 650 nm, while showing a 15% increase around 685 nm and 720 nm. On the other hand, the emission of QDs decreased by a factor of 20 in the QD-PP mixture (Fig. 3c). In order to get rid of the intensity change which might rise due to fluorescence of QD, the emission spectrum of PP-QD was deconvoluted (Fig. 4). It was observed that the emission of QD did not play any role for the rise of intensity of the emission peak around 685 nm and 720 nm (Fig. 4).

**Fig. 5:**
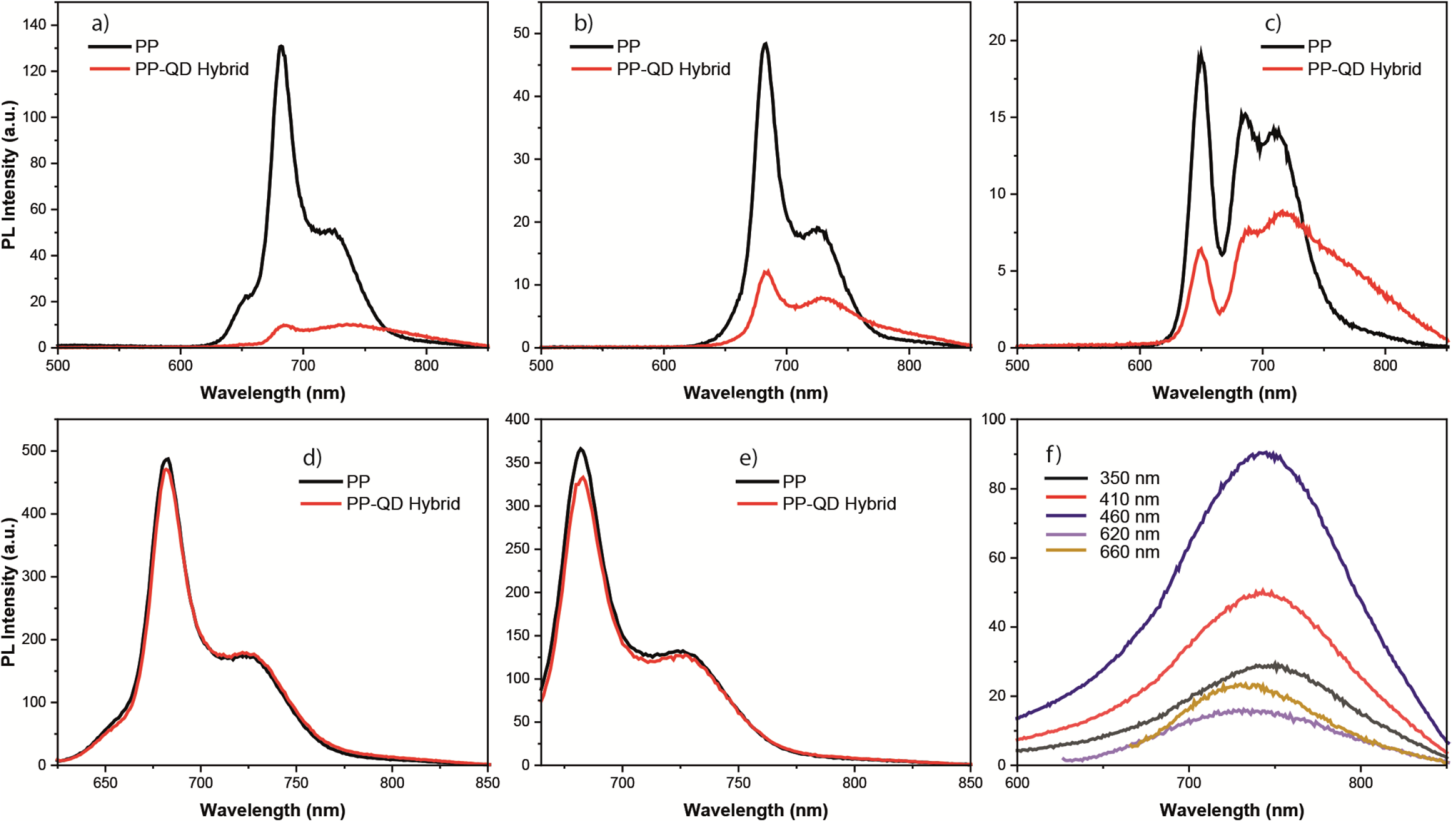
Fluorescence spectrum of photosynthetic pigments (PP) and S-rich CdTeS QD-PP hybrids at excitation wavelengths of **a** 350 nm, **b** 410 nm, **c** 460 nm, **d** 620 nm, and **e** 660 nm. **f** Fluorescence spectrum of S-rich CdTeS QD at various excitation wavelengths

The interaction between PP and S-rich CdTeS QDs exhibited similar results to that of the interaction between PP and CdSeS QDs (Fig. 5). When excited at 350 nm, 95% of PP emission was quenched in the PP-S-rich CdTeS QDs mixture (Fig. 5a). The emission of S-rich CdTeS was still detectable as a shoulder in the wavelength range of 750–850 nm, but with a decreased intensity by 65% upon 350 nm excitation. On the other hand, the PP emission decreased by 75%, while the QD emission decreased by 88% upon 410 nm excitation (Fig. 5b). Similarly, the PP emission decreased by 65%, while the QD emission decreased by 90% upon 460 nm excitation (Fig. 5c). Similar to the observations in the interaction between PP and Te-rich CdTeS QDs, as well as PP and CdSeS QDs, the emission of PP did not exhibit any significant differences in the QD-PP mixture when excited at 620 nm and 660 nm (Fig. 5d and e).

**Fig. 6:**
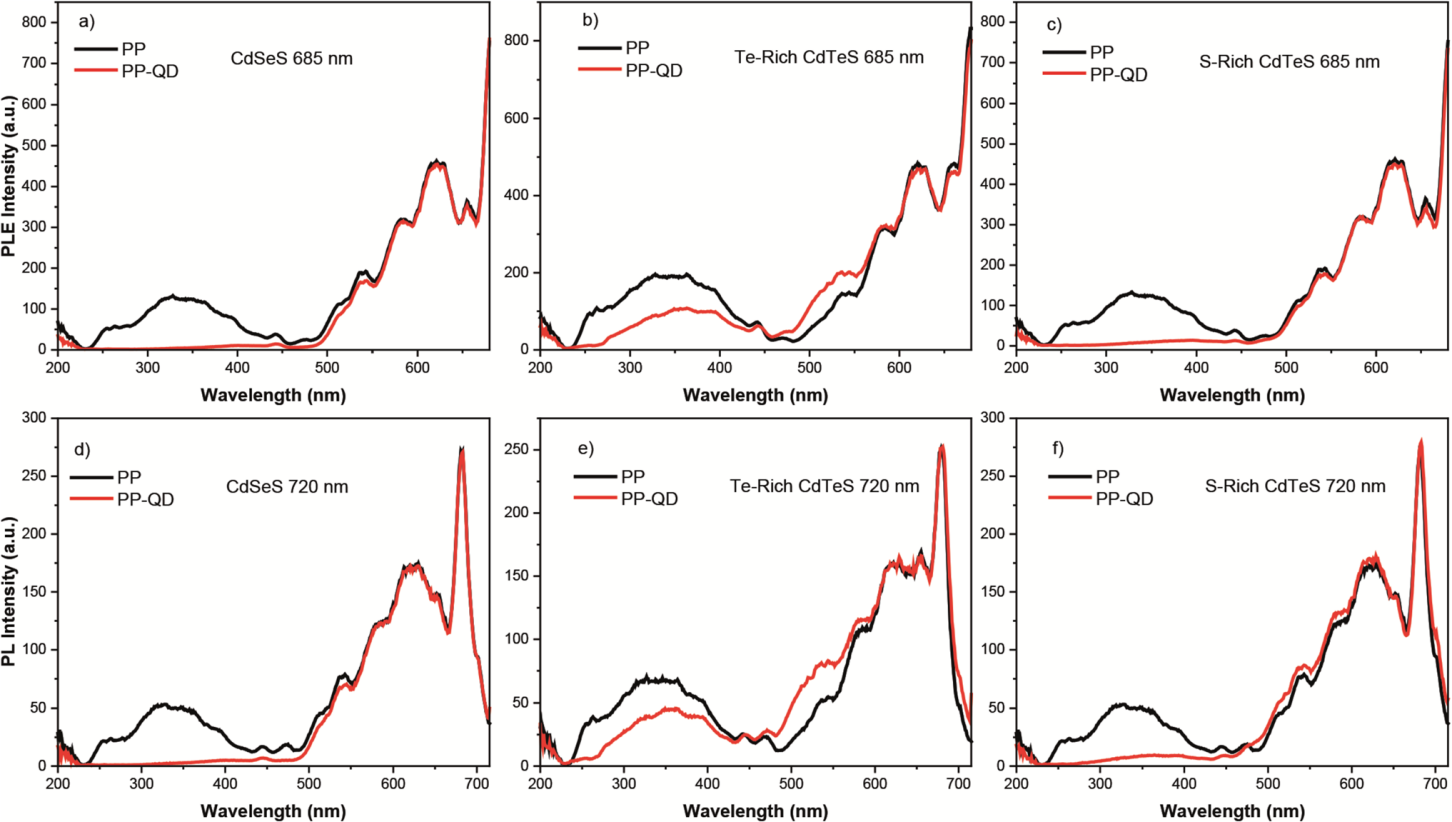
Photoluminescence Excitation spectrum of PP and PP-QD hybrids; **a** QD: CdSeS and emission wavelength of 685 nm, **b** QD: Te-rich CdTeS and emission wavelength of 685 nm, **c** QD: S-rich CdTeS and emission wavelength of 685 nm, **d** QD: CdSeS and emission wavelength of 720 nm, **e** QD: Te-rich CdTeS and emission wavelength of 720 nm, **f** QD: S-rich CdTeS and emission wavelength of 720 nm All spectra were collected at room temperature

To understand the effect of different excitation wavelengths on the emission of PP and PP-QD mixtures, photoluminescence excitation spectrum of each sample was collected. In the photoluminescence excitation spectrum, the wavelength of excitation light is systematically changed, and the luminescence intensity is observed at the typical emission wavelength of the material under investigation. The PLE spectra of QD-PP mixtures were collected at two different emission wavelengths: 685 nm and 720 nm (Fig. 6). Each PLE spectrum of the QD-PP mixture showed a significant difference compared to the PLE spectrum of pure PP (Fig. 6). In the PLE spectrum of the PP-CdSeS QD mixture at both 685 nm and 720 nm emission wavelengths, the PLE band covering the spectrum from 225 to 500 nm completely disappeared (Fig. 6a and d). However, the PLE band covering the spectrum between 500 and 700 nm was identical in both the PLE spectrum of PP and the PLE spectrum of the PP-CdSeS QD mixture (Fig. 6a and 6d). When comparing the PLE spectrum of PP and the PLE spectrum of the PP-Te-Rich CdTeS QD mixture, it was observed that the intensity of the PLE band ranging from 225 to 470 nm decreased by 60% in the PLE spectrum of the PP-Te-Rich CdTeS QD mixture, but it did not disappear completely as in the PLE spectrum of the PP-CdSeS QD mixture (Fig. 6b and e). Conversely, there was a noticeable increase (by 50% on average) in the intensity of the PLE band covering the spectrum between 500 and 575 nm in the PLE spectrum of the PP-Te-Rich CdTeS QD mixture (Fig. 6b and e). It is worth noting that the PLE spectrum of PP and the PLE spectrum of the PP-Te-Rich CdTeS QD mixture were identical in the spectral range of 575 nm to 660 nm (Fig. 6b and e). Comparing the PLE spectrum of PP and the PLE spectrum of the PP-S-Rich CdTeS QD mixture, it was observed that the intensity of the PLE band ranging from 225 to 470 nm disappeared in the PLE spectrum of the PP-Te-Rich CdTeS QD mixture, similar to what was observed in the PLE spectrum of the PP-CdSeS QD mixture (Fig. 6c and f). However, the PLE spectrum of PP and the PLE spectrum of the PP-S-Rich CdTeS QD mixture were identical in the spectral range of 575 nm to 700 nm (Fig. 6c and f).

**Fig. 7:**
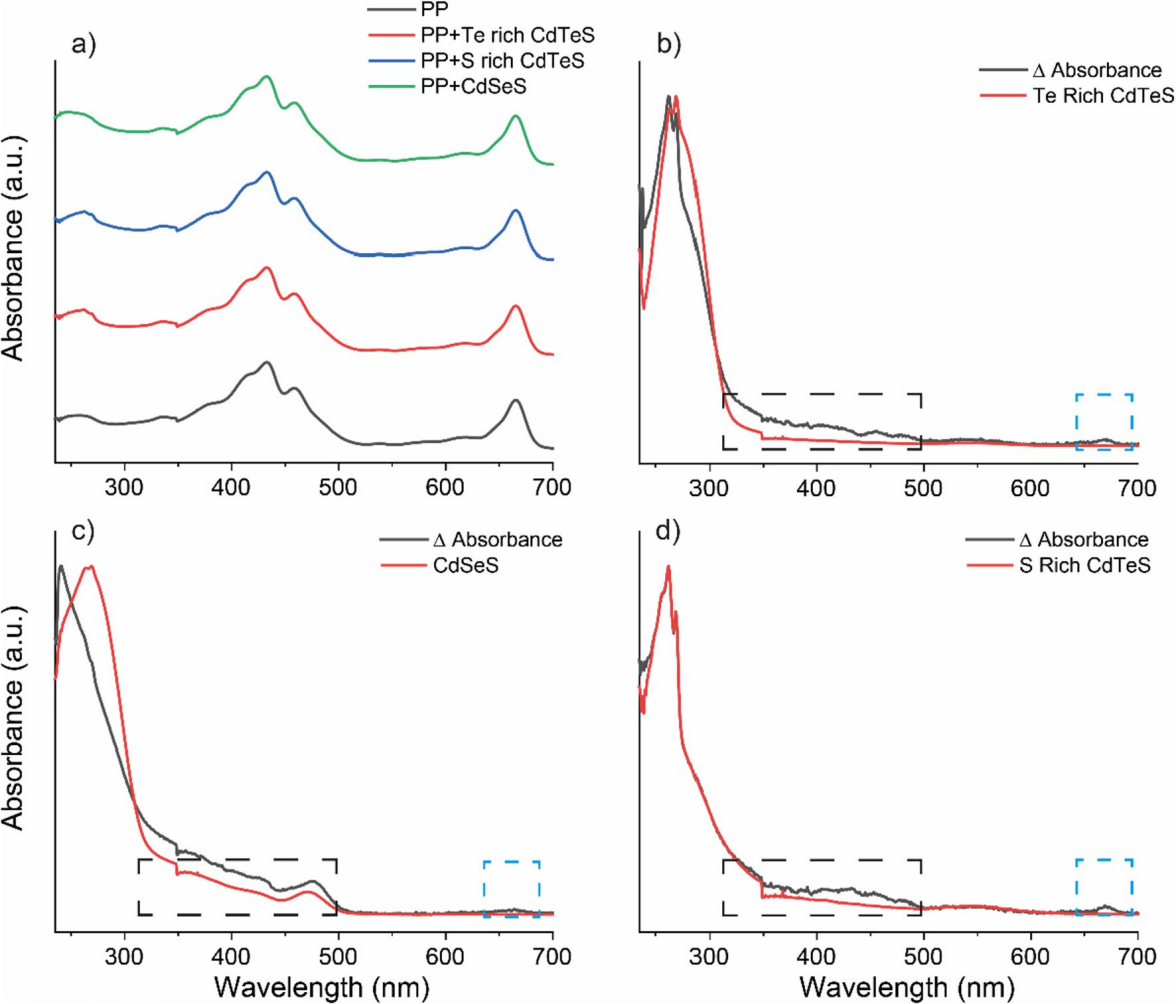
**a** Absorption spectra of PP, PP-Te Rich CdTeS QDs, PP-S Rich CdTeS QDs and PP- CdSeS QDs. ΔAbsorption (PP-QD absorption spectrum—PP absorption spectrum) and absorption spectrum of corresponding QDs; **b** Te Rich CdTeS QDs, **c** CdSeS QDs and **d** S Rich CdTeS QDs

The absorption spectra of PP and PP-QD mixtures were collected for all samples. As QDs were added to the PP mixture, the absorbance spectrum underwent slight alterations; however, the fundamental characteristics of the PP absorption spectrum did not change significantly (see Fig. 7a). To gain a better understanding of the changes in the absorption spectrum of PP-QD mixtures, the absorption spectrum of PP was subtracted from the absorption spectrum of the PP-QD mixture, resulting in the ΔAbsorption spectrum. This was then compared with the absorption spectrum of QD (Fig. 7b, c, and d). Upon comparing the ΔAbsorption spectrum with the absorption spectrum of QDs, it was observed that the ΔAbsorption spectrum closely resembled the absorption spectrum of QD, with slight differences in certain wavelength regions. In each case, the ΔAbsorption spectrum exhibited a broad peak between 350 and 500 nm, as well as a single peak around 665 nm (Fig. 7b, c, and d).

**Fig. 8:**
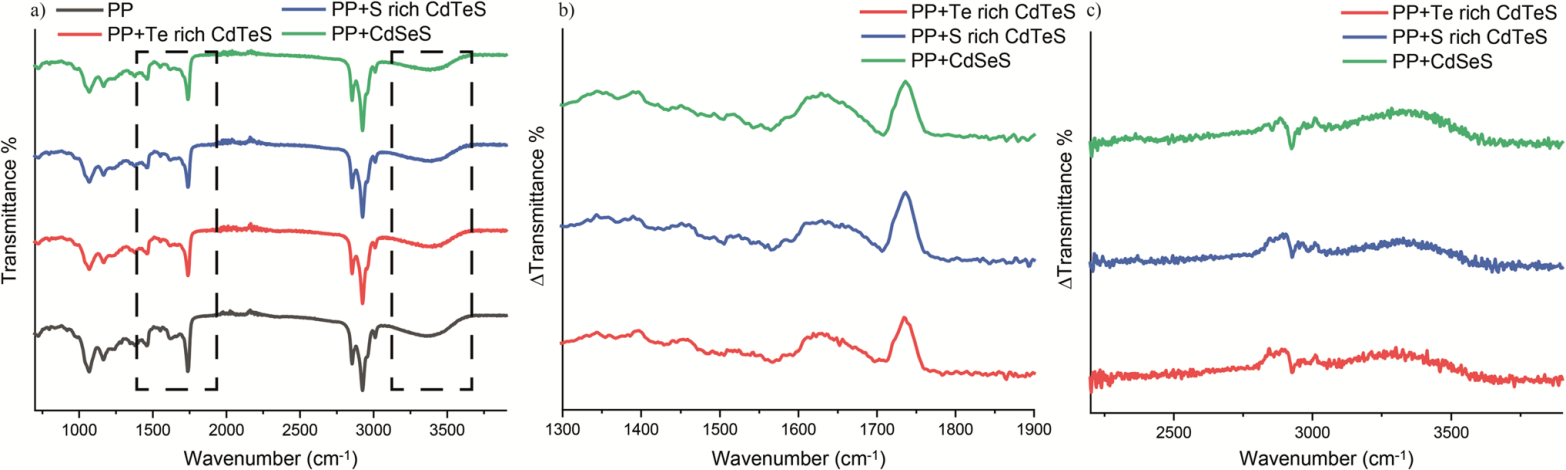
**a** FTIR spectra of PP, PP-Te Rich CdTeS QDs, PP-S Rich CdTeS QDs and PP- CdSeS QDs. The black circle on the right marked the spectral range of 3600 and 3100 cm^−1^ and on the left marked the spectral range of 1900 and 1300 cm^−1^, **b** ΔTransmittance spectrum (PP-QD FTIR spectrum—PP FTIR spectrum) in the spectral range of 1900 and 1300 cm^−1^, **c** ΔTransmittance spectrum (PP-QD FTIR spectrum—PP FTIR spectrum) in the spectral range of 2500 and 3900 cm^−1^

The FTIR spectra of both PP and PP-QD mixtures were recorded as well. The FTIR spectrum of PP exhibited peaks at various bands, including a broad peak between 3600 and 3200 cm^−1^, a triple peak at 3010, 2930, and 2850 cm^−1^, an intense peak at 1710 cm^−1^ followed by a minor peak at 1644 cm^−1^, and a combination of several peaks between 1460 and 1050 cm^−1^ (Fig. 8a). Upon the addition of QDs to the PP solution, the FTIR characteristics of the mixtures closely resembled the FTIR spectrum of PP with slight differences (Fig. 8a). In order to emphasize the minor changes in FTIR spectra, the FTIR spectrum of PP was subtracted from FTIR spectrum of PP-QD conjugates and ΔTransmittance spectrum of each sample was plotted (Fig. 8b, c). Specifically, the intense peak at 1710 cm^−1^, followed by the minor peak at 1640 cm^−1^, decreased slightly (Fig. 8b) and the intensities of the broad peak between 3450 and 3200 cm^−1^ (Fig. 8c) as the PP interacted with QD. However, the remaining FTIR characteristics remained unchanged (Fig. 8a).

The percent change in the quantum yield (QY) of PP at 685 nm emission wavelength after interaction (ΔQY_PP_%) was calculated by modifying following equation (Grabolle et al. 2009);

$${QY}_{x}={QY}_{standart}\frac{{I}_{x}}{{I}_{std}}\times \frac{{OD \, at \, \lambda (exc)}_{std}}{{OD \, at \, \lambda (exc)}_{x}}\times \frac{{n}_{x}^{2}}{{n}_{std}^{2}}$$

where *x* is any fluorophore, *std* is the standard fluorophore with known QY, *I* is the integrated area under the emission peak, OD at λ(exc) is the absorbance at the excitation wavelength, *n* is the refractive index of the solvent. The equation underwent modification based on the following rationale. The PLE spectrum of the samples displayed the emission intensity at specific wavelengths (685 nm or 720 nm) for various excitation wavelengths. Consequently, this spectrum can be employed to compute the QY at a singular emission wavelength, as the emission intensity can be regarded as the area under this specific emission wavelength. Subsequently, the equation was adjusted as follows:

$${QY}_{x}={QY}_{standart}\frac{{I at 685 nm}_{x}}{{I}_{std}}\times \frac{{OD \, at \, \lambda (exc)}_{std}}{{OD \, at \, \lambda (exc)}_{x}}\times \frac{{n}_{x}^{2}}{{n}_{std}^{2}}$$

Then, the ratio of QY of PP-QD conjugate to QY of PP can be calculated for 685 nm emission wavelength by following equation;

$$\frac{{QY}_{PP-QD}}{{QY}_{PP}}=\frac{{QY}_{standart}\frac{{I \, at \, 685 nm}_{PP-QD}}{{I}_{std}}\times \frac{{OD \, at \, \lambda (exc)}_{std}}{{OD \, at \, \lambda (exc)}_{PP-QD}}\times \frac{{n}_{PP-QD}^{2}}{{n}_{std}^{2}}}{{QY}_{standart}\frac{{I \, at \, 685 nm}_{PP}}{{I}_{std}}\times \frac{{OD \, at \, \lambda (exc)}_{std}}{{OD \, at \, \lambda (exc)}_{PP}}\times \frac{{n}_{PP}^{2}}{{n}_{std}^{2}}}$$

Since the solvent is the same for all samples and the standard can be used as the same material for whole samples, this equation can be simplified as;

$$\frac{{QY}_{PP-QD}}{{QY}_{PP}}=\frac{{I \, at \, 685 nm}_{PP-QD}}{{I \, at \, 685 nm}_{PP}}\times \frac{{OD \, at \, \lambda (exc)}_{PP}}{{OD \, at \, \lambda (exc)}_{PP-QD}}$$

And finally;

$$\frac{{QY}_{PP-QD}}{{QY}_{PP}}=\frac{\frac{{I \, at \, 685 nm}_{PP-QD}}{{OD \, at \, \lambda (exc)}_{PP-QD}}}{\frac{{I \, at \, 685 nm}_{PP}}{{OD \, at \, \lambda (exc)}_{PP}}}$$

This term can be calculated by dividing each intensity in the PLE spectrum at every excitation wavelength by the absorbance at each corresponding wavelength in the absorption spectrum.

**Fig. 9:**
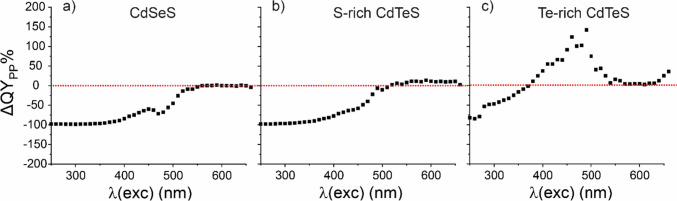
ΔQY_PP_% vs λ(exc) spectrum for **a** CdSeS, **b** S-rich CdTeS and **c** Te-rich CdTeS QDs for 685 nm emission

Then, the change in the QY of PP after conjugation with QD at emission wavelength of 685 nm (ΔQY) can be calculated by subtracting QY of PP from QY of PP-QD conjugate ($\Delta QY={{QY}_{PP-QD}-QY}_{PP}$). Finally, the percent change in QY of PP after interaction with QD (ΔQY_PP_%), which would tell the quantity of enhancement/quenching of emission of PP at 685 nm, can be calculated by following equation;

$$\Delta {QY}_{PP}\%=\frac{\Delta QY}{{QY}_{PP}}\times 100=(\frac{{QY}_{PP-QD}}{{QY}_{PP}}-1)\times 100$$

and plotted against λ(exc);

The quantum yield (QY) of photosynthetic pigments dropped by 100% when exposed to excitation wavelengths ranging from 250 to 400 nm after interacting with CdSeS and S-Rich CdTeS quantum dots (QDs) (Fig. 9a, b). There was an average 50% decrease in the QY of photosynthetic pigments between excitation wavelengths of 250 to 370 nm after interaction with Te-rich CdTeS QDs (Fig. 9c). However, there was no change in the QY of photosynthetic pigments between excitation wavelengths of 500 to 700 nm after interacting with QDs (Fig. 9a, b and c). Notably, the QY of photosynthetic pigments increased significantly, reaching up to 140%, between excitation wavelengths of 420 and 550 nm after interaction with Te-Rich CdTeS QDs (Fig. 9c).

## Discussion

The two-phase synthesis method was chosen as the preferred approach to synthesize the QDs due to two primary reasons (Ünlü 2019; Güleroglu and Ünlü 2021; Kestir et al. 2023). Firstly, the ternary QDs synthesized through two-phase synthesis method exhibit a heterogeneous-gradient structure, with a core enriched in Se or Te, and an outer region rich in S (Ünlü 2019; Güleroglu and Ünlü 2021; Kestir et al. 2023). This unique structure contributes to a high photoluminescence quantum yield (PQY). Secondly, the synthesis process employs mild conditions, such as low temperatures and less toxic cadmium precursors (Ünlü 2019; Güleroglu and Ünlü 2021; Kestir et al. 2023).

The synthesis of the QDs was guided by the absorption spectrum of PPs. CdSeS QDs were synthesized to emit in a spectrum that closely matched the absorption of PPs in the UV region. Te-rich CdTeS QDs were prepared to emit in a spectrum that matched the absorption of PPs in the far red region. Also, S-rich CdTeS QDs were synthesized to emit outside the absorption range of PPs. The main purpose of synthesizing CdSeS and Te-rich CdTeS QDs was to check the possibility of energy transfer from QDs to PPs. Furthermore, the synthesis of S-rich CdTeS QDs aimed to investigate the outcomes when there is no possibility for energy transfer between the QDs and PPs.

The XRD peaks of the QDs had broad peaks at observed angles, indicating the nano-size nature of QDs (Ünlü 2019; Güleroglu and Ünlü 2021; Kestir et al. 2023). The size of the QDs was determined using the Scherrer rule, resulting in dimensions of 2.4 nm for CdSeS, 2.7 nm for CdTeS, and 3.2 nm for S-rich CdTeS, which were also in agreement with TEM results of each QD. The structure of the Cd chalcogenide QDs was determined by analyzing the shifts observed in the [111] peak compared to bulk cubic crystals of CdSe, CdS and CdTe. The chemical composition of CdSeS was found to be CdSe_0.18_S_0.82_, Te-Rich CdTeS was CdTe_0.19_S_0.81_, S-Rich CdTeS was CdTe_0.07_S_0.93_. It should be noted that the concentration of the S precursor was always higher than the concentration of the Se or the Te precursor, which led to presence of a high amount of S in each quantum dot (Ünlü 2019; Güleroglu and Ünlü 2021; Kestir et al. 2023).

The absorption and PLE spectra of raw PP collected at two different wavelengths, 685 nm and 720 nm, displayed typical soret and Q bands of chlorophyll a and b (Ünlü et al. 2014).

All QDs were coated with the same surfactant (oleic acid) with the aim of eliminating the effect of functional groups present on the surface to the possible energy transfer mechanisms. The absence of –OH peaks in the FTIR spectra of the QDs indicated the binding of oleic acid to the Cd-based QDs through the –OH groups. The rest of the oleic acid peaks was observed as was observed in literature before, suggesting that oleate form was still intact (Caruntu et al. 2015; Masuku et al. 2021). In conclusion, it was observed that the Cd-based QDs exhibited distinct compositions and photophysical properties while maintaining identical surface properties.

To investigate the impact of the interaction between QDs and PPs on their emission, five different excitation wavelengths were utilized, each serving a distinct purpose. The choice of each excitation wavelength was carefully made based on specific considerations.

At 350 nm, the excitation wavelength allowed for the simultaneous excitation of both QDs and PPs. However, it is worth noting that QDs efficiently absorb light around 350 nm and convert absorbed light into fluorescence with a high quantum yield (Erkan et al. 2023; Kestir et al. 2023). By employing the 350 nm excitation wavelength, the potential for energy transfer between QDs and PPs was examined. It should be noted that QDs could be excited with 410 nm and 460 nm light as well, but the emission of QDs decreased by at least 75% (CdTeS) or disappeared (CdSeS) upon excitation with 620 nm and 660 nm excitation.

The selection of the remaining excitation wavelengths was based on the absorption spectra of chlorophyll a (chl a) and chlorophyll b (chl b) (Dall’Osto et al. 2014). Excitation at 410 nm and 660 nm was specifically chosen to excite chl a, while excitation at 460 nm and 620 nm was selected to target chl b (Dall’Osto et al. 2014). This deliberate selection of wavelengths aimed to investigate the impact of QDs on the photophysical properties of chl a and chl b separately, while these two molecules remained intact within a protein complex. The emission peaks of chl a and chl b mostly overlap, yet the chl b possesses a typical emission peak at 650 nm (Dall’Osto et al. 2014). Thus, the appearance of the peak at 650 nm upon excitation with 460 nm and 620 nm served as evidence of the selective excitation of chl b at these specific wavelengths (Dall’Osto et al. 2014).

Significant differences were observed when comparing the emission characteristics of PP in PP-QD mixtures at 350 nm excitation, the wavelength at which QDs were mostly excited. The emission of PP was quenched by 100% in the PP—CdSeS QD mixture, quenched by 95% in PP—S-rich CdTeS QD mixtures, and quenched by %65 in the PP—Te-rich CdTeS QD mixture. These results indicated that the chemical composition of QD structure had a significant impact on the interaction between PP and QD. When PP was mixed with CdSeS QDs, PP could not exhibit any radiative electron transition. Furthermore, the emission intensity of CdSeS QDs decreased by 90%, indicating a reduction in the amount of radiative electron transitions in CdSeS and PP upon their interaction. On the other hand, emission of PP was still observable in PP—CdTeS interactions. In the case of PP—Te-rich CdTeS QD interaction, the emission intensity of PP decreased by 65%, suggesting that the radiative electron transition in PP was not completely blocked but still slightly affected. PP was able to use the 350 nm light to induce radiative electron transitions in the presence of Te-rich CdTeS QD and thus the chlorophyll emission could be observed. Nevertheless, the decrease in emission intensity of PP and Te-rich CdTeS QDs indicated an effective interaction between PP and Te-rich CdTeS QDs. As the content of S increased in CdTeS QDs, the intensity of PP emission in the PP-QD mixture quenched by 95%. These observations suggest that as the Te amount in QD decreased and the thickness of the S-rich outer surface increased, the number of radiative electron transitions decreased in terms of the emission of PP and QDs.

When mixtures of PP and QDs were excited with chl a excitation wavelengths (410 nm and 660 nm), each mixture displayed different results. The emission of PP was quenched by 80% in the PP-CdSeS QD mixture, was quenched by 75% in PP-S-rich CdTeS QD mixtures, and quenched by 50% in the PP-Te-rich CdTeS QD mixture upon excitation at 410 nm. When PP was mixed with CdSeS QDs, PP exhibited radiative electron transition with a significant decrease in intensity. Additionally, the emission intensity of CdSeS QDs decreased by 96%, indicating a reduction in the amount of radiative electron transitions in both CdSeS and PP upon their interaction. The interaction between PP and S-rich CdTeS QDs was similar to that of PP-CdSeS QD interaction; PP emission decreased by 88%, yet remained detectable. Both results indicated that the spectral response of PP at 410 nm was significantly reduced in the presence of CdSeS and S-rich CdTeS QDs, suggesting that PP could still function in the presence of these QDs, albeit with a lower spectral response. A similar conclusion could be drawn for the PP-Te-rich CdTeS QD interaction; however, it should be noted that PP displayed a significantly higher amount of radiative transitions, indicating that spectral response of PP was still efficient at 410 nm excitation wavelength in the presence of Te-rich CdTeS. These results indicated that as PP was excited at chl a excitation wavelengths, spectral response of PP could be modified to a significant extent, which could be regulated by using different types of QDs. On the other hand, the emission of PP in the PP-QD mixture upon 660 nm excitation was identical to that of the pure PP sample, suggesting that the presence of QD did not affect the spectral response of PP at higher wavelengths. This observation could be explained by two possible simultaneous mechanisms: 1) The structure of PP remained intact and was not damaged by the presence of QD, and 2) QD could not absorb light above 600 nm, therefore it could not affect the spectral response of PP at higher wavelengths.

As observed in the emission of PP in mixtures with QDs excited at chl a excitation wavelengths, different results were obtained when mixtures of PP and QDs were excited with chl b excitation wavelengths of 460 nm and 620 nm. In the PP-CdSeS QD mixture, the emission of PP was significantly quenched by 75% upon excitation at 460 nm, while in the PP-S-rich CdTeS QD mixture, it was quenched by 65% at the same excitation wavelength. These results indicate that when PP was mixed with CdSeS QDs, a significant decrease in the intensity of radiative electron transition of PP was observed. Moreover, the emission intensity of CdSeS QDs also significantly decreased by 80%, indicating a reduction in the amount of radiative electron transitions for both CdSeS and PP upon their interaction. A similar conclusion can be drawn for the interaction between PP and S-rich CdTeS QDs. However, it is important to note that PP exhibited a significantly higher amount of radiative transitions, suggesting that spectral response of PP was still observable at the 460 nm wavelength in the presence of S-rich CdTeS QDs. Interestingly, the emission intensity of the PP-Te-rich CdTeS QD mixture increased significantly in the 675–735 nm band and remained unchanged around the 650 nm band. When the emission spectrum of the PP-QD mixture was deconvoluted and the emission peak of the unquenched QD was extracted, it was calculated that the intensity of the emission band in the 675–735 nm range increased by 40%. These results indicate that the spectral response of PP should increase by at least 40% upon 460 nm excitation. On the other hand, the emission of PP in the PP-QD mixture upon 620 nm excitation was identical to that of the pure PP sample. This suggests that the presence of QDs did not affect the spectral response of PP at higher wavelengths, providing additional evidence consistent with the observations made at the 660 nm excitation wavelength.

The PLE spectra of raw PP and PP-QD mixtures were compared to understand how the presence of QDs affects the spectral response of PP at two different emission wavelengths: 685 nm and 720 nm. In the fluorescence spectrum of PP, the wavelengths of 685 nm and 720 nm corresponded to the mirror images of the main Q_y_(0,0) absorption band and its satellite Q_y_(1,0) band of chlorophyll, respectively. In the PLE spectra of the PP-CdSeS mixture, the soret bands between 225 and 500 nm completely disappeared for both 685 nm and 720 nm. On the other hand, the Q bands in the PLE spectra of PP and PP-CdSeS mixture were identical. A similar observation was made for the PP-S-rich CdTeS QDs, with the only difference being that the soret band of PP was slightly observable in the mixture. These observations indicated that the spectral response of PP at the soret absorption bands was nullified in the presence of CdSeS and S-rich CdTeS QDs. However, in the PLE spectrum of the PP—Te-rich CdTeS QD mixture, the radiative transition raised upon excitation in soret absorption band of chlorophylls did not completely disappear. Moreover, the intensity of the PLE spectrum of PP—Te-rich CdTeS significantly enhanced by 50% (on average) between 500 and 575 nm, both at 685 nm and 720 nm. This wavelength range corresponds to the green color in the light spectrum, which is generally not absorbed by photosynthetic organisms. It was also observed that as PP interacted with Te-rich CdTeS QDs, spectral response of PP upon green light excitation significantly increased, and the energy was effectively transferred to chlorophylls. It should be noted that the PLE spectra of raw PP and the PP—Te-rich CdTeS QD mixture were identical in the 575 nm to 700 nm region.

To comprehend the mechanisms underlying the interaction between PP and QDs and to assess the stability of PP following this interaction, a comparison of the absorption and FTIR spectra of PP and PP-QD mixtures were conducted. The characteristics of absorption and FTIR spectrum of PP were also observed in absorption and FTIR spectrum of PP-QD mixtures. These observations revealed that the structure of PP remained stable after the interaction. However, there were subtle variations in both the absorption and FTIR spectra following the interaction. Traditionally, minor differences in the absorption spectrum of chlorophyll have been regarded as evidence of interaction with another molecule or ion (Gaines et al. 1964; Grajek et al. 2020). In our study, the absorbance spectrum of PP and PP-QD exhibited notable differences in the same region as the absorbance spectrum of QDs, resembling the absorbance characteristics of QDs. Nevertheless, additional differences were observed in two regions: between 350 and 500 nm and around 665 nm. These distinctions indicated that QDs were interacting with chlorophylls, primarily inducing alterations in the spectral response of chlorophylls. The alterations or shifts observed at these specific wavelengths were commonly associated with interactions among chlorophyll molecules via formyl or vinyl groups (Silva et al. 2022). Consequently, the variations in the absorption spectrum of PP-QD conjugates indicate that the interaction between QDs and PP primarily occured through the formyl groups of chlorophylls. This interaction affects the energy transitions of chlorophylls, as evidenced by the changes in the soret and Q bands of the absorption spectrum of PP following its interaction with QDs. The disparities in the FTIR spectra of PP and PP-QD mixtures were primarily noted in the 3450–3200 cm^−1^ range and at the peaks of 1710 cm^−1^ and 1640 cm^−1^. These specific wavenumbers correspond to amine, ester C=O, and keto C=O respectively (Wang et al. 2004), suggesting that QDs approached chlorophylls primarily through C=O bonds and influenced the vibrational states of amine groups in the center, leading to a change in the spectral response of PP.

**Fig. 10:**
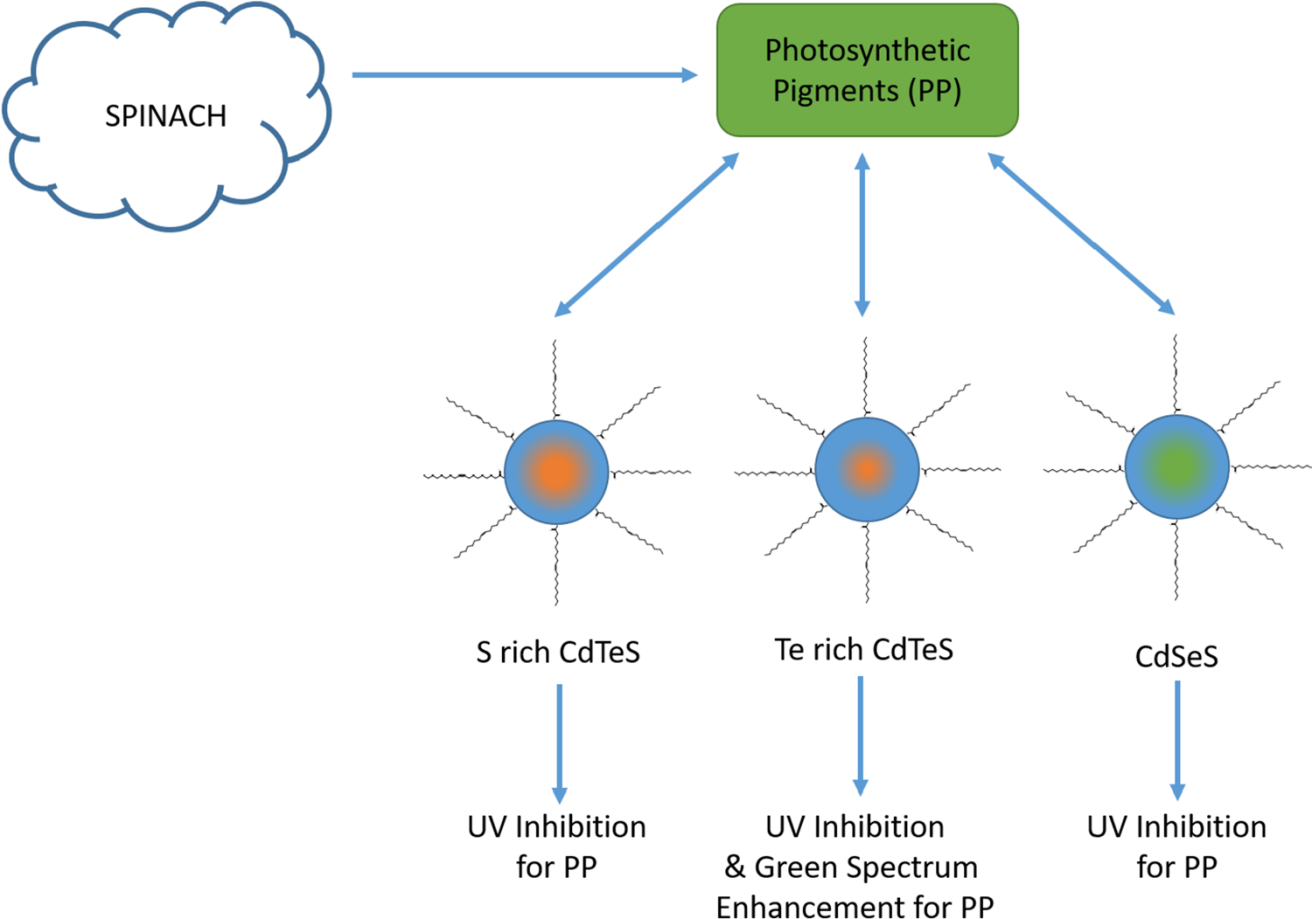
Schematic illustration of effect of quantum dots on PP

The alterations in the spectral response of PP in the presence of QDs were examined by observing the change in the quantum yield QY of PP emission at 685 nm. When exposed to CdSeS and S-rich CdTeS QDs, the QY of PP emission notably decreased (up to 100%) within the 250–400 nm excitation wavelength range. Interestingly, the absorption spectrum of PP remained unaffected in the presence of QDs, indicating a substantial reduction in radiative transitions and an enhancement of non-radiative processes. In contrast, within the 500–660 nm excitation range, there was no significant alteration in the QY of PPs. Moreover, the QY of PP experienced a substantial increase (up to 140%) within the 420–550 nm excitation wavelength range in the presence of Te-rich CdTeS QDs. These results suggested that the spectral response of PP can be adjusted by utilizing different types of QDs (Fig. 10).

## Conclusion

In conclusion, a strategic two-phase synthesis method was employed to fabricate QDs based on CdSeS, CdTeS, and S-rich CdTeS, emphasizing their interaction with raw PP. This approach yielded QDs with a heterogeneous-gradient structure, resulting in a high photoluminescence quantum yield, while the synthesis conditions, featuring mild temperatures and less toxic precursors, enhanced the method's suitability. The subsequent uniform coating of QDs with oleic acid ensured stable surface properties. The investigation into the interaction between QDs and PPs, under various excitation wavelengths, demonstrated that the chemical composition of QDs significantly influenced the spectral response of PPs. Additionally, the selective excitation of chlorophyll a and b revealed distinct responses, indicating the potential to modulate the spectral characteristics of PPs through tailored QD synthesis. Further analysis of absorption and FTIR spectra, along with quantum yield measurements, provided insights into the stability of PPs and the mechanisms of interaction with QDs. Overall, this comprehensive study not only elucidated the intricate interplay between QDs and PPs but also highlighted the potential for fine-tuning the spectral response of photosynthetic systems through the judicious selection of quantum dot compositions.

## Data Availability

Data is available upon request.
